# Sentinel monitoring of COVID-related daily activity in primary care practices of the canton of Vaud

**DOI:** 10.1093/eurpub/ckac131.560

**Published:** 2022-10-25

**Authors:** Y Mueller, D Auderset, M Maeder, J Schwarz, E Masserey

**Affiliations:** 1 Department of Family Medicine, Center for Primary Care and Public Health, Lausanne, Switzerland; 2 Cantonal Health Directorate, Lausanne, Switzerland

## Abstract

**Introduction:**

During the COVID pandemic, COVID-related data collected in family medicine were scarce. We aimed to monitor cantonal trends of COVID-related activity in family medicine and paediatric practices during the year 2021.

**Methods:**

Family physicians and paediatricians established in the canton of Vaud were invited to join an ad hoc sentinel surveillance system. Online data collection was based on daily activity reports and monthly questionnaires. In particular, participants categorized daily counts of consultations and phone calls into predefined COVID-related categories.

**Results:**

Thirty-seven practices contributed regularly to the system between March 20th and December 31st 2021. Out of 81'407 medical consultations, 4'950 (6.1%) were related to new COVID suspicions as defined by the Federal Office of Public Health, and 5'252 (6.4%) otherwise related to COVID. Depending on the week and the practice, between 5.6% and 26.5% of face-to-face consultations were COVID-related. In paediatrics, COVID-related activity corresponded mostly to new COVID suspicions (11.2% of on-site consultations), whereas among family physicians other COVID topics predominated (9.8% of face-to-face consultations), mainly questions about vaccination. Consultations for persisting COVID-related symptoms were stable at a low level throughout the year, and constituted less than 1% of all consultations. Most practices swabbed patients for SARS-CoV-2 tests, and an increasing proportion performed rapid antigenic tests over the year. In paediatrics, COVID-suspicions were not systematically tested.

**Conclusions:**

Throughout 2021, COVID-related consultations constituted an important part of family medicine and paediatric practices’ activity in the canton of Vaud. Monitoring COVID-related activity in primary care during a pandemic documents how physicians translate recommendations into practice and provides health authorities with valuable information to guide public health action.

**Key messages:**

• Throughout 2021, COVID-related consultations constituted an important part of family medicine and paediatric practices’ activity in the canton of Vaud.

• Monitoring COVID-related activity in primary care during a pandemic documents how physicians translate recommendations into daily practice.

